# Co-Amorphous Systems Based on Dihydroquercetin and l-Lysine: Synthesis and Evaluation

**DOI:** 10.3390/pharmaceutics17121528

**Published:** 2025-11-27

**Authors:** Artem A. Svotin, Maria D. Korochkina, Anastasia A. Khodyachikh, Diana R. Kolesnikova, Amir Taldaev, Eduard V. Bocharov, Alexander V. Dzuban, Andrey N. Utenyshev, Gennadii V. Shilov, Youyan Zeng, Bo Li, Roman P. Terekhov, Irina A. Selivanova

**Affiliations:** 1 Nelyubin Institute of Pharmacy, Sechenov First Moscow State Medical University, Trubetskaya Str. 8/2, 119991 Moscow, Russia; 2 Laboratory for the Study of Single Biomacromolecules, Institute of Biomedical Chemistry, Pogodinskaya Str. 10/8, 119121 Moscow, Russia; 3 Laboratory of Biomolecular NMR-Spectroscopy, Shemyakin-Ovchinnikov Institute of Bioorganic Chemistry, Miklukho-Maklaya Str. 16/10, 117997 Moscow, Russia; 4 Research Center for Molecular Mechanisms of Aging and Age-Related Diseases, Moscow Center for Advanced Studies, Kulakova Str. 20/1, 123592 Moscow, Russia; 5 Department of Chemistry, Lomonosov Moscow State University, Leninskiye Gory 1-3, 119991 Moscow, Russia; 6 Federal Research Center of Problems of Chemical Physics and Medicinal Chemistry RAS, Academician Semenov Ave. 1, 142432 Chernogolovka, Russia; 7 Guangdong Metabolic Diseases Research Center of Integrated Chinese and Western Medicine (Institute of Chinese Medicine), MOE Key Laboratory of Glucolipid Metabolic Disorder, Guangdong TCM Key Laboratory for Metabolic Diseases, Guangdong Pharmaceutical University, Wai Huan Dong Road 280, Guangzhou 510006, China

**Keywords:** dihydroquercetin, flavonoid, l-lysine, water-soluble, cytotoxicity, nuclear magnetic resonance, differential scanning calorimetry, thermogravimetry, scanning electron microscopy, X-ray powder diffraction

## Abstract

**Background/Objectives**: Dihydroquercetin (DHQ), also known as taxifolin, is a natural flavonoid which has anti-inflammatory and wound-healing biological effects. One of the main limitations for developing formulations with DHQ is its low solubility in water at room temperature. One of the high-potential co-formers for increasing its solubility is l-lysine, which has an aliphatic amino group in the side radical capable of entering into intermolecular interactions with the phenolic hydroxyl groups of DHQ. **Methods**: Several modifications were obtained using grinding, drying, and lyophilization methods. Subsequent evaluation was conducted using a combination of physicochemical and biological analytical methods. **Results**: Obtained modifications could be described as very easily soluble substances. The absence of the formation of new covalent bonds between the compounds during the formation of such systems was established. The glass transition effect was detected at 64 °C for the obtained films. It is important to note that as a result of studying the cytotoxic properties of the objects, a decrease in cytotoxicity was established during lyophilization of the mechanical mixture of the initial components. For these lyophilizates, the IC50 value was 0.025 mg/mL, 0.068 mg/mL, 0.145 mg/mL, and 0.288 mg/mL for the 3T3, HEK293, Caco-2, and HUVEC cell lines, respectively. **Conclusions**: Co-amorphous systems of DHQ and l-lysine in the form of films and lyophilizates were obtained and described. These objects may be interesting from the point of view of increasing the solubility of natural flavonoids, which solves one of the main problems in developing drugs based on them.

## 1. Introduction

**Figure 1:**
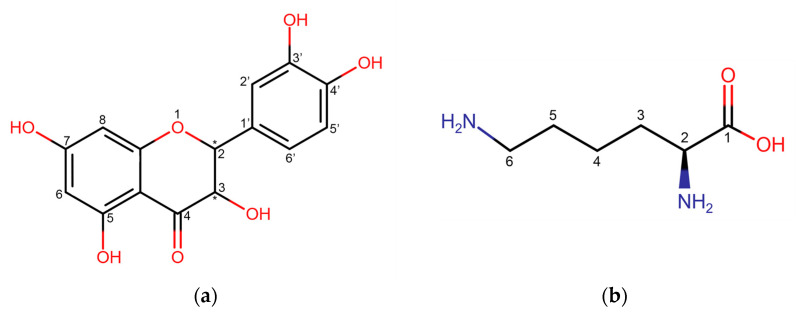
Structural formula of (**a**) dihydroquercetin; (**b**) l-lysine. * show the position of stereocenters.

Dihydroquercetin (DHQ) – 2,3-dihydro-3,5,7-trihydroxy-2-(3,4-dihydroxyphenyl)-4*H*-1-benzopyran-4-one – also known as taxifolin (Figure 1a), is a natural flavanonol, the main raw material base of which is the wood of Siberian larch (*Larix sibirica* Ledeb.) and Dahurian larch (*Larix dahurica* Turcz.) [1]. This compound has demonstrated anti-inflammatory [2,3] and wound-healing [4,5] biological effects. These effects are attributed not only to its pronounced antioxidant activity [6,7] but also to its ability to regulate the expression of genes such as CD68, CD31, and VEGF [8]. Additionally, there is evidence of DHQ activating the AMPK/Nrf2/HO-1 signaling pathway in macrophages, which contributes to the reduction in inflammatory processes [9].

One of the main limitations in developing DHQ-based drugs is its low water solubility at room temperature. According to the European Pharmacopoeia 11.5 [10], its solubility is classified as “very slightly soluble”. This results in limited bioavailability [1], requiring modifications to enhance its suitability for medicinal formulations. Current research primarily focuses on increasing DHQ’s solubility, which in turn improves its bioavailability. Previously explored methods include lyophilization from acetonitrile and methanol solutions [11] and the formation of inclusion complexes with cyclodextrins [12,13]. However, these methods have several limitations, including the limited solubility of the resulting products and the complexity of the technology, which complicates their transfer to industrial production.

Among the approaches aimed at increasing the bioavailability of flavonoids, co-crystallization and co-amorphization [14,15,16] stand out, as they combine ease of implementation with a significant increase in solubility. This makes them an advanced field for further research. One of the most promising co-formers is the amino acid l-lysine (Figure 1b), which contains an aliphatic amino group in its side chain capable of forming intermolecular interactions with the acidic phenolic hydroxyl groups of DHQ. Moreover, amino acids have already been used to produce water-soluble compositions with other flavonoids. For example, the solubility of genistein increased from 5 × 10^−5^ ± 1 × 10^−5^ mg/mL for the unmodified flavonoid to 1.919 ± 0.161 mg/mL and 0.938 ± 0.014 mg/mL when combined with lysine and arginine, respectively [17]. Additionally, lysine has demonstrated anti-inflammatory activity by reducing the levels of TNF-α, IL-8, and MIF [18].

Thus, the aim of this study was to develop water-soluble compositions of DHQ and lysine and to characterize their properties. The assessment of the obtained objects was carried out using a range of physicochemical and biological methods of analysis. This allowed us to do a comprehensive characterization and description of the modifications.

## 2. Materials and Methods

### 2.1. Materials

DHQ (95% purity) and state standard reference sample of DHQ (99.99% purity) were purchased from “Ametis” JSC (Blagoveshchensk, Russia). l-lysine monohydrate (98.5–101.0% purity, pharma grade) was provided by NeoFroxx GmbH (Darmstadt, Germany). Needle-modified Dulbecco’s Modified Eagle Medium (DMEM), minimum essential medium (MEM), trypsin solution with ethylenediaminetetraacetate (EDTA), and penicillin/streptomycin (P/S) solution were received from Gibco (Thermo Fisher Scientific Inc., Waltham, MA, USA); fetal bovine serum (FBS) was from ExCell Bio Group (Shanghai, China). Also, 99.9% dimethyl sulfoxide (DMSO) was supplied by Beyotime Inc. (Shanghai, China). Cell counting kit (CCK-8) was provided by Selleck Chemicals LLC (Houston, TX, USA). Biosharp (Hefei, China) provided 0.01 M phosphate-buffered saline (PBS).

### 2.2. Preparation of Mechanical Mixture, Films, and Lyophilizates

To obtain a mechanical mixture (DHQ/LYS_mix_), 3.04 g of DHQ (DHQ_raw_) was mixed with 3.28 g of l-lysine monohydrate (LYS_raw_), which corresponds to a molar ratio of 1:2, stirred and ground in a mortar for 15 min.

To form films (DHQ/LYS_film_), 5.0 g of the resulting mechanical mixture was dissolved in 10.0 mL of distilled water and applied to a solid polyethylene substrate, kept in a drying chamber FED 53 (Binder GmbH, Tuttlingen, Germany) for 10–15 min at a temperature of 65 °C. The resulting films were detached from the substrate.

To obtain a DHQ lyophilizate (DHQ_lyo_), 2.0 g of DHQ was dissolved in 150.0 mL of distilled water at 80 °C. The resulting solution was subjected to rapid freezing at −78 °C for 24 h, after which the flask was connected to a freeze dryer BK-FD12P (Biobase, Jinan, China) operating for 36 h under the following conditions: −56 °C, 1 kPa.

To obtain the l-lysine and mechanical mixture lyophilizates (LYS_lyo_ and DHQ/LYS_lyo_, respectively), 5.0 g of substances were dissolved in 50.0 mL of distilled water at room temperature. The resulting solution was subjected to rapid freezing at −78 °C for 24 h, after which the flask was connected to a freeze dryer operating for 36 h under the following conditions: −56 °C, 1 kPa.

All prepared modifications were summarized in Table 1.

### 2.3. Solubility Studies

The analysis was performed in accordance with the pharmacopoeial monograph of the State Pharmacopoeia of the Russian Federation XV, which is equivalent to the terms of the Pharmacopoeia of the Eurasian Economic Union and the European Pharmacopeia 11.5 at ambient conditions.

A measured amount of solvent was added to the suspension of the substance, and the mixture was continuously shaken for 1 min and placed in a thermostat set at a temperature of 24.5–25.5 °C for 15 min. If the substance had not completely dissolved, the shaking process was repeated for another minute, and the solution was kept in the thermostat for another 15 min period at the same temperature.

### 2.4. Physicochemical Analysis

#### 2.4.1. Attenuated Total Reflection–Fourier Transform Infrared Spectroscopy (ATR-FTIR)

FTIR spectra were recorded in the range of 400–4000 cm^−1^ with a resolution of 4 cm^−1^ using an FSM 2202 (Infraspek Ltd., St.-Petersburg, Russia) equipped with a diamond ATR crystal. Background subtraction was performed, and each spectrum was recorded with 10 scans.

#### 2.4.2. X-Ray Powder Diffraction (XRPD)

Powder diffraction profiles were obtained on an AERIS X-ray diffractometer (Malvern, Panalytical, Almelo, The Netherlands), equipped with a vertical wide-angle goniometer and a Peltier solid-state detector. A Cu (copper) cathode (λ = 1.54 Å) was the radiation source, current intensity was 15 mA, and voltage was 40 kV. Data were collected at 295 K in the 2*θ* range from 5° to 50° with a step size of 0.012° and a charge accumulation time of 7 s. Each sample was placed inside the plastic sample holder in the same way.

#### 2.4.3. Differential Scanning Calorimetry (DSC)

DSC analysis was carried out on a DSC 204 F1 Phoenix^®^ differential scanning calorimeter (NETZSCH, Selb, Germany). Samples weighing 2.00–5.00 mg were tested in aluminum crucibles with pierced lids under dry nitrogen flow (70 mL·min^−1^) with a heating rate of 10 °C·min^−1^. The instrument was previously calibrated for temperatures and enthalpies of phase transitions of pure (99.999%) standard substances in compliance with ASTM practices E967 [19], E968 [20], and E2253 [21]: cyclohexane, Hg, Ga, benzoic acid, In, Sn, Bi, Pb, Zn, and CsCl. The average calibration error was 5% for the heat and 0.2 K for the temperature. Experimental data were processed in NETZSCH Proteus^®^ Software (Version 8.0.3) according to ASTM E794 [22] and ISO 11357-1 [23].

#### 2.4.4. Thermogravimetry (TG)

TG analysis was performed on a TG 209 F1 Libra^®^ thermobalance (NETZSCH, Selb, Germany). Samples weighing 5.00–10.00 mg were tested in alumina crucibles (lid with a hole) under dry nitrogen flow (20 mL·min^−1^) with a heating rate of 10 °C·min^−1^. The instrument was previously calibrated for temperatures of phase transitions of pure (99.999%) standard substances in compliance with ASTM E1582 [24] practice: In, Sn, Bi, Zn, Al, Ag, and Au. Calcium oxalate monohydrate was used for the validation of thermobalances. The systematic error in measuring mass loss did not exceed 1%. Experimental data were processed in NETZSCH Proteus^®^ Software according to ASTM E2550 [25].

#### 2.4.5. ^1^H Nuclear Magnetic Resonance (^1^H-NMR)

For NMR analysis, the samples (10 mg each) were dissolved in 100% D_2_O. The solutions were placed in a 5 mm NMR tube. NMR spectra of ^1^H were acquired at 298 K on an 800 MHz Bruker Avance III NMR spectrometer (Bruker BioSpin, Rheinstetten, Germany) equipped with a TXI triple resonance probe.

### 2.5. Scanning Electron Microscopy (SEM)

SEM was performed on a JSM-6320LA (“JEOL” Ltd., Tokyo, Japan) SEM machine with a W electron source that worked at a pressure of 5 × 10^−7^ atm. The diameter of the sample holders was 32 mm. An IB-3 ion counter (“EIKO Corporation” Ltd., Tokyo, Japan), operated at 8 mA and 0.16 Torr, was used for the spraying of Au, which was carried out for 3 min, ensuring a layer thickness of 15 nm, avoiding electric currents and sticking of the picture. Magnification values varied from ×30 to ×2000, so the influence of gold layer thickness was negligible.

### 2.6. Cytotoxicity Assay

#### 2.6.1. Cell Culture Conditions

The cells were cultured in a medium containing DMEM:FBS:MEM:P/S in a ratio of 88:10:1:1 for Caco-2 (immortalized human colorectal adenocarcinoma) cells, and DMEM:FBS:P/S in a ratio of 89:10:1 for HUVEC (Human umbilical vein endothelial cells), 3T3 (mouse embryonic fibroblasts), and HEK293 (immortalized human embryonic kidney) cell lines. The cell cultures were maintained in 100 mm diameter Petri dishes within a CelMate^®^ CO_2_ Incubator (Esco Lifesciences Group, Singapore) at 37 °C in an atmosphere containing 5% carbon dioxide and a relative humidity of 95%. The culture medium was refreshed every 24 h.

#### 2.6.2. Assessment of Cell Concentration

To determine cell concentration, 1.0 mL of a trypsin solution containing EDTA, pre-warmed to 37 °C, was added to a Petri dish and incubated for up to 5 min. The trypsinization was stopped by adding 1.0 mL of nutrient medium, followed by rinsing the inner surface of the Petri dish with the same suspension. The resulting cell suspension was centrifuged at 1000 rpm for 3 min. The supernatant was removed, and the cell pellet was resuspended in 10.0 mL of nutrient medium. Subsequently, 100 µL of the solution was introduced into the Neubauer chamber XB.K.25 (QUIJING, Shanghai, China), and the concentration (cells/mL) was calculated using the following formula:

(1) $$C=\frac{N_{cell}}{N_{squares}}\times 10^{3},$$

where $N_{cell}$ represents the number of cells counted in the Neubauer chamber field and $N_{squares}$ represents the number of squares in the Neubauer chamber used for concentration calculation.

#### 2.6.3. Assessment of Cytotoxicity

The cell suspension was diluted with culture medium to a concentration of 40,000 cells/mL and dispensed at 100 µL per well into a 96-well plate. Then, 100 µL of the analyzed samples, dissolved in DMEM, was added to the cell suspension at concentrations ranging from 10.0000 mg/mL to 0.0188 mg/mL. In addition, several wells were designated as “positive” and “negative” controls. For these controls, a doubled amount of cell suspension and a doubled amount of nutrient medium were added to the wells, respectively.

The mixtures were incubated for 24 h at 37 °C in an atmosphere containing 5% carbon dioxide and a relative humidity of 95%. After the specified incubation period, 10 µL of CCK-8 reagent was added to each well, followed by incubation for an additional 1 h under identical conditions.

The resulting solutions were analyzed on a microplate spectrophotometer Eon (BioTek, Winooski, VT, USA) at the maximum absorption of the reduced form of CCK-8, measured at 450 nm. The optical density values were then used to calculate cell viability (*V*) according to the formula:

(2) $$V=\frac{D_{n}-D_{0}}{D_{100}-D_{0}}\times 100\%$$

where $D_{n}$ is the optical density of the analyzed sample, $D_{0}$ is the optical density of the negative control, and $D_{100}$ is the optical density of the positive control.

The obtained values were subjected to regression analysis to develop a linear regression model of the dependence of cell survival on the concentration of the analyzed sample. Based on these linear relationships, the half-maximal inhibitory concentration (IC50) for each analyzed sample was calculated as the concentration at which cell viability is 50%.

#### 2.6.4. Statistical Processing

The blank experiment was performed three times for each concentration of the analyzed sample of each study object. Using the obtained data, arithmetic means and the confidence interval (*p* = 0.05) were calculated. Subsequently, linear regression models of the test solution optical density dependence on the concentration of the analyzed sample were constructed.

Cytotoxicity was assessed in an octuplicate for each concentration of each analyzed study object sample for each cell line. Using the obtained data, arithmetic means and a confidence interval (*p* = 0.05) were calculated. Subsequently, linear regression models were constructed to describe the relationship between the optical density of the test solution and the concentration of the analyzed sample.

To assess significant differences in the observed cytotoxicity and to substantiate the presence of synergy or inhibitory effects between the components of the multicomponent study objects, a two-way analysis of variance (ANOVA) was performed based on the optical density values of the test samples (α = 0.05).

In addition, the effect of interaction between the components of the multicomponent objects of study was illustrated using isoboles reflecting the concentration of each multicomponent object’s individual component at which IC50 is expected to be achieved if an additive effect is observed between the components.

### 2.7. In Vitro Dissolution Study

#### 2.7.1. Dissolution Conditions

In vitro dissolution was performed using a DT Light Series Dissolution Tester DT 126 (Erweka GmbH, Haan, Germany) equipped with a paddle and containing 900 mL of distilled water as the dissolution medium. The system was run at a speed of 50 rpm and maintained at 37.0 °C ± 0.5 °C. An amount of 100 mg of DHQ_raw_ or 208 mg of DHQ/LYS_mix_, DHQ/LYS_lyo_, and DHQ/LYS_film_ (equivalent to 100 mg of DHQ_raw_) was introduced into the vessel. At time points of 1, 5, 15, 30, and 60 min, samples of 1 mL were taken; the withdrawn volume was replaced with an equivalent volume of fresh medium. The samples were then filtered through a 0.22 μm Nylon Welded Syringe Filter (Labfil, Hangzhou, China) and analyzed by high-performance liquid chromatography (HPLC). Results are presented as a mean value ± standard deviation of three replicates of each sample.

#### 2.7.2. Chromatographic Conditions

The chromatographic system LicArt 62 (Labconcept LLC, St. Petersburg, Russia) consisted of a QP-62d pump, T-85C thermostat, spectrophotometric detector UV-62, auto sampler S-103dc, and an Inspire C18 5 μm 150 × 4.6 mm column (Dikma Technologies Inc., Foothill Ranch, CA, USA). The mobile phase consisted of solvents A—distilled water with trifluoroacetic acid (pH 2.4)—and B—acetonitrile. Elution was conducted at 35 °C in isocratic mode, with 70% of A and 30% of B and a flow rate of 0.35 mL/min. The injection volume was 20.0 µL. Detection was carried out by a UV-detector at a wavelength of 288 nm, which corresponds to the absorption maximum of DHQ.

To construct a calibration curve, 50.0 mg of the state standard reference sample of DHQ and 54.0 mg of recrystallized LYS_raw_ (equivalent to a 1:2 mole ratio) were dissolved in 100 mL of distilled water. A series of 9 consecutive dilutions with a concentration of DHQ in the range from 0.0001 to 0.125 mg/mL was prepared from the resulting stock solution. Results are presented as a mean value ± standard deviation of three replicates of each sample.

## 3. Results and Discussion

### 3.1. Determination of Optimal Conditions for Obtaining Objects

The molar ratio of DHQ_raw_ and LYS_raw_ was set at 1:2, as the main focus of the study was based on the solubility of the resulting mixtures. When LYS_raw_ was added in a ratio lower than 1:1 compared to DHQ, the resulting mixture could be described as “soluble” according to the European Pharmacopoeia 11.5. Once this ratio was reached, the solubility increased and began to exceed 1 g/mL. Therefore, in order to achieve a highly soluble mixture and minimize the use of LYS, a molar ratio of 1:2 was selected for further study.

DHQ_raw_ thermal analysis data was used to select the optimal drying temperature for the DHQ/LYS_film_ preparation solution. Based on the maximum of the first endothermic effect, which occurred at 89 °C, a temperature below this value was chosen, which reduces drying time compared to room temperature.

### 3.2. Visual Description

**Figure 2:**
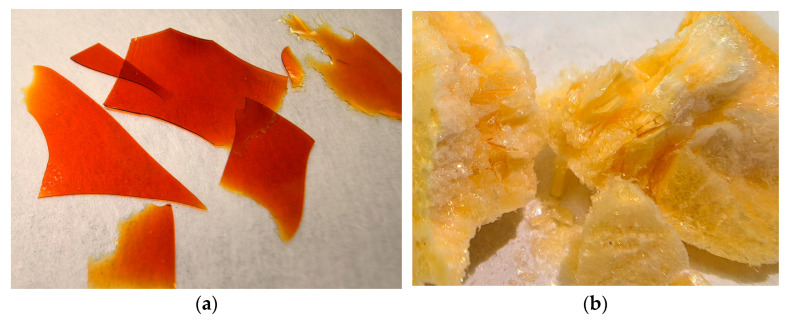
Photographic images of obtained objects: (**a**) films of dihydroquercetin and l-lysine; (**b**) lyophilizate of mechanical mixture of dihydroquercetin and l-lysine.

DHQ/LYS_film_ could be characterized as a glass-like, fragile (approximately as ice) material. The color varied from yellow to red depending on the thickness of the layer, with orange being the most commonly observed color (Figure 2a).

Obtained DHQ/LYS_lyo_ was lightweight, foam-like, with a porous structure and a scabrous yet shiny surface. The color varied from bright to light-yellow (Figure 2b).

### 3.3. Solubility

DHQ/LYS_film_ and DHQ/LYS_lyo_ were characterized as “very easily soluble” in water at room temperature. This compared favorably with the initial flavonoid, which demonstrated being “very slightly soluble” under similar conditions. In general, the problem of increasing the solubility and bioavailability of flavonoids has been actively studied recently [26]. One of the main methods proposed for DHQ is the production of inclusion complexes with cyclodextrins and their modifications [12,27,28]. The obtained structures are characterized by a significant increase in the flavonoid solubility after encapsulation. The method for producing phospholipid nanoparticles of DHQ was also developed [29]. The study on Wistar rats showed that the modification has 2 times greater bioavailability when taken orally than the initial flavonoid.

### 3.4. Structure of the Solid Phase

**Figure 3:**
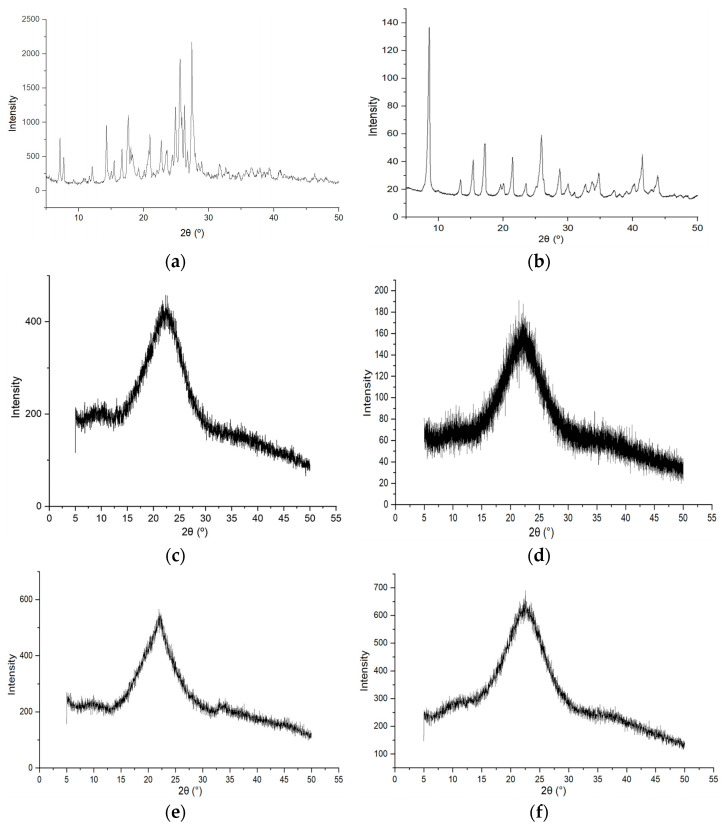
Diffraction profiles of the studied objects: (**a**) dihydroquercetin substance; (**b**) l-lysine monohydrate substance; (**c**) lyophilizate of mechanical mixture of dihydroquercetin and l-lysine; (**d**) films of dihydroquercetin and l-lysine; (**e**) lyophilizate of mechanical mixture of dihydroquercetin and l-lysine after 9 months of storage at room conditions; (**f**) films of dihydroquercetin and l-lysine after 9 months of storage at room conditions.

The diffraction pattern (Figure 3a) of DHQ_raw_ was characterized by a set of peaks with the following 2*θ*: 7.2°, 7.7°, 12.8°, 14.3°, 15.0°, 15.5°, 16.7°, 17.6°, 18.0°, 18.3°, 19.2°, 20.1°, 21.0°, 22.7°, 23.5°, 24.4°, 24.9°, 25.6°, 26.3°, 26.7°, 27.4°, 28.4°, 28.9°, and 31.7°.

In the LYS_raw_ profile (Figure 3b) there were peaks with following 2*θ*: 8.6°, 13.4°, 15.4°, 17.2°, 19.6°, 20.0°, 21.4°, 23.5°, 25.9°, 28.7°, 30.0°, 31.0°, 32.7°, 33.7°, 34.7°, 37.1°, 40.2°, 41.5°, and 43.8°.

These diffraction patterns showed that the initial compounds have a crystalline structure. These data are consistent with previous studies that have shown that the initial flavonoid [11,30] and the amino acid [31] have a crystalline structure.

The amorphous halo on the diffraction profile of DHQ/LYS_lyo_ (Figure 3c) reached its maximum at 2*θ* of 22.3°.

The diffraction pattern of the DHQ/LYS_film_ (Figure 3d) was characterized by the presence of an “amorphous halo”, with a maximum at 2*θ* of 22.2°.

Thus, during the production of DHQ/LYS_film_ and DHQ/LYS_lyo_, similar processes occurred, leading to the creation of amorphous products with a similar structure. This was confirmed by the similarity in the maximum at 2*θ* in both objects. So, as a result, the co-amorphous systems of DHQ and l-lysine were obtained. Both objects remained stable, amorphous structures when stored under room conditions for 9 months (Figure 3e,f).

Co-amorphous systems are widely used to increase the bioavailability and solubility of various compounds [32]. In this case, proteinogenic amino acids are often considered as co-formers for these structures [33,34]. A co-amorphous system of carvedilol with the aspartic and glutamic acids was obtained by the spray-drying method [35]. It was shown that the formation of this structure is associated with acid–base interactions between the components. Also, a number of co-amorphous systems based on griseofulvin with amino acids were obtained by grinding in a ball mill [36]. As a result, an increase in the stability and solubility of the original compound was demonstrated, especially for tryptophan. These data demonstrate the high potential of amino acids as co-formers for the modification of poorly soluble compounds.

### 3.5. Chemical Structure of the Obtained Objects

To establish the structure of the obtained objects, they were studied using ATR-FTIR and ^1^H NMR methods.

The ATR-FTIR spectra of the initial compounds corresponded to the literature data on characteristic absorption bands [37,38]. During the lyophilization of DHQ_raw_, a smoothing of the spectrum in the “hydrogen bond region” was observed, which may be associated with an increase in hydrogen bonding during the amorphization of the original crystalline substance. In this case, formed hydrogen bonds can be both intermolecular and intramolecular, involving interactions between the phenolic hydroxyl group (OH5) and C=O, as well as between the alcoholic hydroxyl group (OH3) and C=O [39]. Similar spectral changes were observed during the amorphization process of indomethacin [40], which was carried out using co-grinding with Neusilin US2 and melting methods. Comparable changes occur during the process of lyophilization of l-lysine.

The DHQ/LYS_mix_ exhibited absorption bands characteristic of both original components. However, a pronounced smoothing in the “hydrogen bond region” was observed, likely due to the formation of hydrogen bonds between the amino groups of the amino acid and the phenolic hydroxyl groups of the flavonoid. This was confirmed by visual observation during grinding: The mixture began to acquire a yellowish-orange hue, which could be caused by hydrogen bond formation or ionization of the phenolic hydroxyl groups of DHQ. No new absorption bands were detected, indicating that no covalent bonds formed during the grinding process.

No pronounced changes were observed in the spectra of the DHQ/LYS_lyo_ and DHQ/LYS_film_ compared to the DHQ/LYS_mix_. This suggests the formation of similar structures without the creation of new covalent bonds between the components. All spectral differences were confined to the “hydrogen bond region,” which may be attributed to variations in moisture content among the samples [41]. All the original ATR-FTIR spectra are presented in Figure S1.

Taking into account the XRPD and ATR-FTIR data, it could be said that the DHQ/LYS_lyo_ and the DHQ/LYS_film_ were similar substances in their chemical nature. For this reason, some of the further analysis methods were performed only on the films.

To confirm the absence of new covalent bond formation during the formation of films, they were analyzed using ^1^H NMR (Table 2).

As a result of the NMR spectra analysis, the absence of signals of all DHQ’s phenolic hydroxyl groups in the DHQ/LYS_film_ was established. All other signals from the protons of DHQ and l-lysine remained unchanged or shifted slightly. These data confirm the assumptions about the possibility of forming hydrogen bonds between the initial components and the absence of new covalent bond formation.

### 3.6. Thermal Analysis

**Figure 4:**
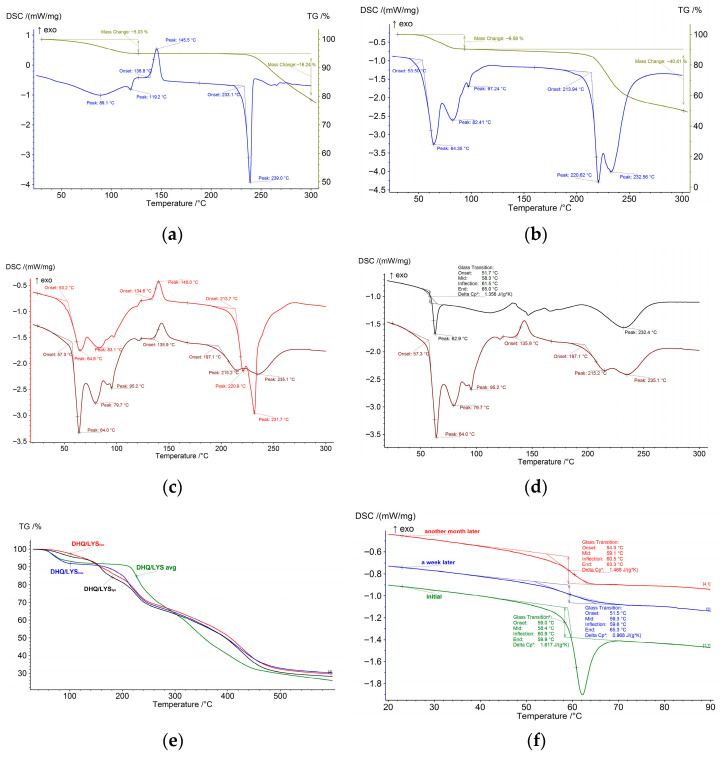
Thermograms of the studied objects: (**a**) dihydroquercetin substance; (**b**) l-lysine monohydrate substance; (**c**) mechanical mixture of dihydroquercetin and l-lysine (brown) and calculated differential scanning calorimetry curve of dihydroquercetin substance and l-lysine monohydrate substance mixture in 1:2 molar ratio (red); (**d**) mechanical mixture of dihydroquercetin and l-lysine (brown) and films of dihydroquercetin and l-lysine (black); (**e**) mechanical mixture of dihydroquercetin and l-lysine (blue), films of dihydroquercetin and l-lysine (red), lyophilizate of mechanical mixture of dihydroquercetin and l-lysine (black) and calculated differential scanning calorimetry curve of dihydroquercetin substance and l-lysine monohydrate substance mixture in 1:2 molar ratio (green); (**f**) differential scanning calorimetry curves of films of dihydroquercetin and l-lysine during aging. * denotes calculated values.

The DSC curve of DHQ_raw_ (Figure 4a) starts with a broad endothermic effect up to 130 °C with a peak at 89 °C, accompanied by slow mass loss on the TG curve due to elimination of the water from the sample, which exists in the form of monohydrate [42]. The exothermic effect at 139 °C relates to the crystallization of the amorphous part of DHQ_raw_ [43]. Further heating leads to the sample melting (233 °C) and the following decomposition.

The DSC curve of LYS_raw_ (Figure 4b) has two bold endothermic effects over the range of 40–100 °C, accompanied by mass loss on the TG curve. The consequent transition from monohydrate to hemihydrate and then to the anhydrous form of lysine occurs [44]. At 214 °C, lysine melts with further decomposition.

The experimental DSC curve of the DHQ/LYS_mix_ (Figure 4c) is almost identical to the superposition of individual DSC curves of pure substances in the same ratio, indicating there is no interaction between them in this form.

At the same time, the effects corresponding to lysine dehydration partly disappear on the DSC curve of DHQ/LYS_film_ (Figure 4d), and the observed line suggests the presence of a glass transition-type transformation. The rest of the DSC curves look similar. Apparently, water removal occurs during sample preparation, which is also evidenced by a lower mass loss at the first stage (up to 100 °C) on the TG curves of DHQ/LYS_film_ and DHQ/LYS_lyo_ (Figure 4e).

To study the nature of the transition at 60 °C, the sample of DHQ/LYS_film_ was subjected to thermal cycling with continuous aging at room temperature—reheating after a week and after a month (Figure 4f). As was shown, T_mid_ remains almost the same, and the heat capacity change approaches the initial value, so the cumulative effect is revealed and the glass transition is established. The glass transition was previously shown also for flavonoid naringenin after spray-drying, where it had a temperature of around 93 °C [45].

### 3.7. SEM

**Figure 5:**
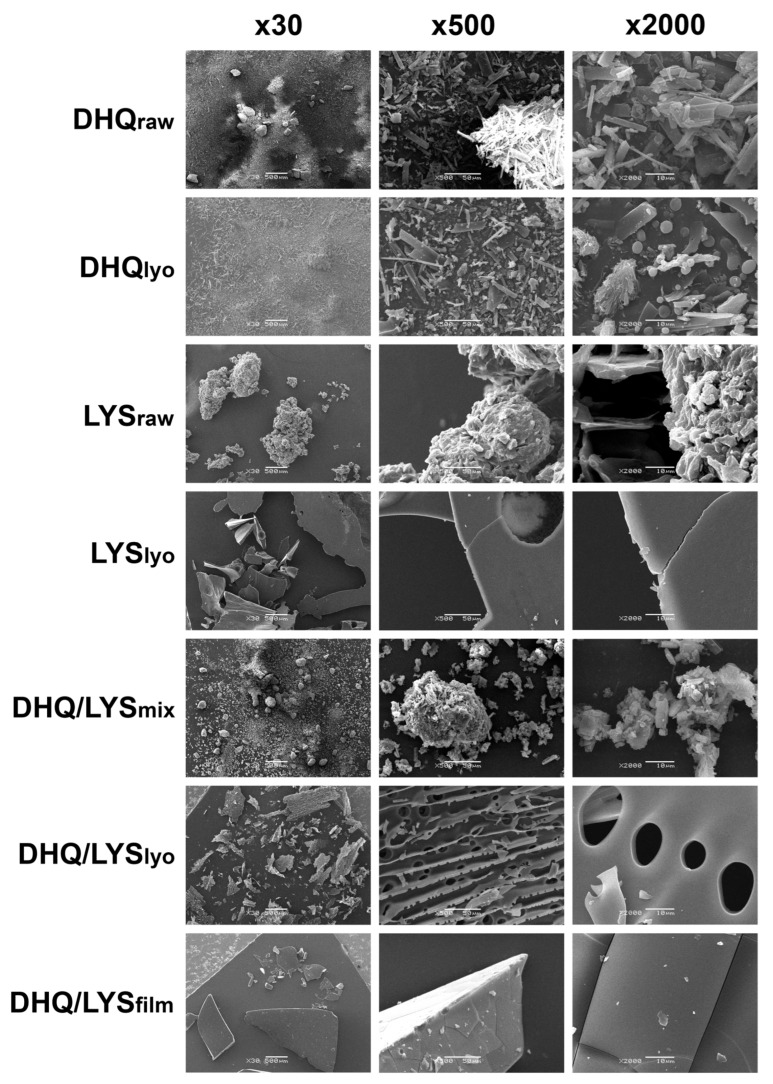
Scanning electron microscopy images of obtained objects.

A closer observation of DHQ_raw_ (Figure 5) shows it looked like independent, sharp-edged, and crystal-like sticks of different sizes, approximately 21.0 ± 6.7 nm by length and 5.2 ± 2.7 nm by width. At ×30 magnification it could be clearly seen that the particles are congregated into bigger pieces (165.0 ± 42.1 × 125.0 ± 33.5 nm).

In the DHQ_lyo_ sample, the sticks became narrower and longer: 33.0 ± 14.0 and 6.5 ± 2.5 nm by length and width, respectively. The emergence of solid spherical elements and hollow ones that look like beads could be clearly seen at ×2000 magnification. The diameter of the spheres varied around 3.0 ± 0.8 nm, with 1 nm perforations for the hollow ones; the surface was rather smooth. Some shaggy elements (16.1 ± 4.4 and 7.7 ± 2.9 nm by length and width, respectively) and thin slices with 1.6 ± 0.4 nm holes with sleek outline also appeared.

LYS_raw_ was characterized as smoothed-out shrunken “islands” of size from 131.25 ± 78.4 × 125.0 ± 84.9 to 808.3 ± 351.0 × 475.0 ± 185.5 nm, formed from stuck-together 12.4 ± 4.0 × 11.3 ± 3.6 nm pieces.

The LYS_lyo_ represented plain, smooth-surfaced objects from 470.0 ± 80.0 × 300.0 ± 102.8 to 1480.0 ± 589.3 × 650.0 ± 212.5 nm that looked half-transparent like glass, with rough, uneven edges. There were also some uneven holes (90.0 nm in diameter) encountered on the surface.

The DHQ/LYS_mix_ appearance was between that of DHQ_raw_ and LYS_raw_: it consisted of lysine-like “islands” (197.5 ± 72.5 and 145.0 ± 29.9 nm length and width, respectively) of stuck-together particles with many DHQ_raw_-like 22.5 ± 3.9 × 14.4 ± 5.4 nm pieces. The edges of the elements were smooth, closer to LYS_raw_. Nevertheless, at ×30 magnification, the mix looked more like DHQ_raw_.

As for DHQ/LYS_lyo_, the “islands” had come apart, becoming thin and plain, 565.0 ± 273.7 × 280.0 ± 94.9 nm, perforated all over the surface. The smooth-edged holes had different sizes: The small ones (3.5 ± 1.0 nm in diameter) were located on the other side of a big-holed (13.5 ± 2.9 nm in diameter) area, on a perpendicularly disposed surface. Notably, most of the small holes were incomplete, cut to a half, which could be observed at ×500 magnification.

The DHQ/LYS_film_ was abruptly different in appearance: the elements looked more like 555.0 ± 388.0 × 409.0 ± 257.4 nm pieces of glass, which had a transparent surface with straight cracks (44.5 ± 8.8 nm by length) and plain cut at the edges, with tiny (2.0 ± 0.8 nm diagonally) chipped particles all over the surface.

### 3.8. Cytotoxicity Assessment

The cytotoxicity of the DHQ samples and their reducing ability with respect to CCK-8 in a blank experiment were evaluated in the concentration range from 2.50 mg/mL to 0.02 mg/mL. For DHQ_raw_, a linear relationship was observed between the optical density of the test sample and the concentration of the added study object from 0.02 mg/mL to 1.25 mg/mL. The regression coefficient was 0.9895 (Figure S2). This suggests the applicability of this linear regression model for predicting the contribution of the analyzed object’s reducing ability to the observed optical density within the indicated concentration range. The obtained results indicate a pronounced reducing ability of DHQ.

For l-lysine and its compositions with DHQ, a linear relationship was observed between the test sample optical density and the amount of the added object of study. The regression coefficients for LYS_lyo_, DHQ/LYS_mix_, and DHQ/LYS_lyo_ were 0.9583, 0.9984, and 0.9990, respectively. This further indicates the applicability of this linear regression model for predicting the contribution of the reducing ability of the analyzed object to the observed optical density within the indicated concentration range.

When assessing cytotoxicity, a pronounced inverse linear relationship was observed between the natural logarithm of cell survival and the concentration of the introduced sample, as evidenced by the regression coefficients, which ranged from 0.9171 for DHQ/LYS_mix_ on the HEK293 cell model to 0.9981 for LYS_lyo_ on the HUVEC cell line (Figure S3). In most cases, the IC50 value fell within the designated concentration range, allowing a reliable calculation of its value. The only exception, where the value of this parameter was above the maximum concentration, was for LYS_lyo_ on the 3T3 model. These data were excluded from further analysis.

For DHQ_raw_, the IC50 values were 0.039, 0.065, 0.088, and 0.096 mg/mL for the HEK293, Caco-2, HUVEC, and 3T3 cell models, respectively. The obtained data are consistent with the literature [46,47]. For LYS_lyo_, the IC50 values were 0.538, 0.826, and 1.490 mg/mL for Caco-2, HUVEC, and HEK293 cell line models, respectively. DHQ/LYS_mix_ was characterized by less-pronounced cytotoxicity than DHQ_raw_: The minimum IC50 value was found in the Caco-2 cell model (0.067 mg/mL), and the maximum value was found in HUVEC (0.254 mg/mL). For the 3T3 and HEK293 cell lines, the IC50 values were 0.105 and 0.088 mg/mL, respectively. For DHQ/LYS_lyo_, the IC50 value was 0.025 mg/mL, 0.068 mg/mL, 0.145 mg/mL, and 0.288 mg/mL for the 3T3, HEK293, Caco-2, and HUVEC cell lines, respectively.

ANOVA analysis revealed a significant effect of lyophilization on the cytotoxicity of DHQ_raw_ and LYS_raw_ samples in relation to HEK293 (*p* = 0.02655), 3T3 (*p* = 1.1 × 10^−7^), and Caco-2 (*p* = 0.00089) cells. In the case of the first two models, cytotoxicity potentiation was observed, while in the third, inhibition occurred.

**Figure 6:**
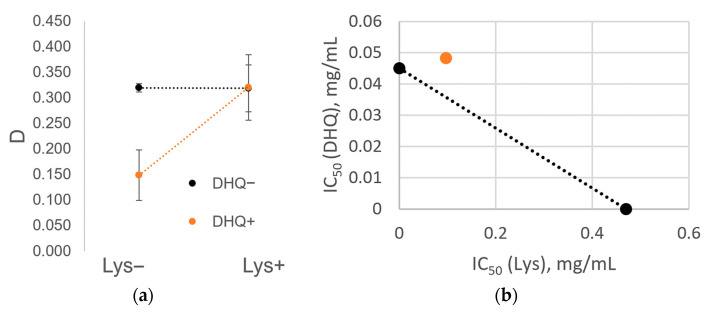
Inhibitory effect on cytotoxicity of lyophilizate of mechanical mixture of dihydroquercetin and l-lysine in relation to Caco-2 cells from the simultaneous presence of dihydroquercetin and l-lysine: (**a**) The ratio of the adjusted optical densities depending on the presence of dihydroquercetin or l–lysine at a concentration of 0.015 mg/mL in the test sample; (**b**) the IC50 value of lyophilizate of mechanical mixture of dihydroquercetin and l-lysine (orange dot) lies above the isobole of its components.

The inhibitory effect on cytotoxicity of DHQ/LYS_lyo_ in relation to Caco-2 cells, from the simultaneous presence of DHQ and l-lysine (Figure 6a), represents the ratio of adjusted optical densities depending on the presence of both compounds at a concentration of 0.015 mg/mL in the test sample. The non-parallel lines on the adjusted optical densities graph indicate an interaction between DHQ and l-lysine under the action of the lyophilizate on Caco-2 cells (parallel lines would indicate the absence of interaction). Figure 6b shows that the IC50 of DHQ/LYS_lyo_ (orange dot) lies above the isobole of its components. It shows pairs of concentrations of DHQ and l-lysine, represented as dots on the isobologram (a dotted line depicting a specific effect with the intersection points of the abscissa and ordinate axes). It represents combined concentrations that are additive when both components exhibit a constant effectiveness ratio. Given that the set IC50 value is reached at higher concentrations, with the lyophilizate point located above the isobologram, the combination of concentrations indicates subadditivity (cytotoxicity is inhibited).

Before definitively concluding that a subadditive effect exists, ANOVA analysis is necessary to determine whether the visual manifestation of inhibition is sufficiently large compared to random variations in the data. The results of this analysis are presented in Table 3. The actual value of the Fisher ratio exceeds its critical value for the interaction of the components (*p* = 2.5 × 10^−11^), allowing us to reject the null hypothesis of the absence of inhibition and confirm the significance of the observed subadditive effect.

### 3.9. In Vitro Dissolution

An in vitro dissolution test was conducted to evaluate the dissolution profile of DHQ from the obtained samples. In order to quantify the flavonoid content in the dissolution medium, a quantitative analysis method based on HPLC was developed. The calibration curve R^2^ = 0.9999 and RSD values obtained for each point confirmed the suitability of using this method for this purpose (Figure S4 and Table S1).

The choice of a sample weight equivalent to 100 mg of DHQ is based on current in vivo research in this field. Single doses used in these studies contain the flavonoid at a level not exceeding the selected value [48,49,50,51,52].

**Figure 7:**
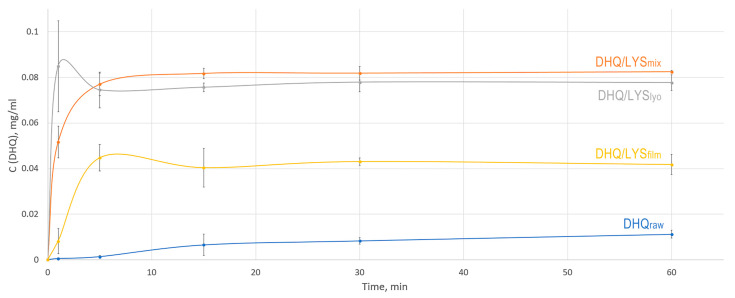
In vitro dissolution profiles of obtained objects: blue—dihydroquercetin substance; yellow—films of dihydroquercetin and l-lysine; gray—lyophilizate of mechanical mixture of dihydroquercetin and l-lysine; orange—mechanical mixture of dihydroquercetin and l-lysine.

As a result, we found that DHQ/LYS_lyo_ showed the highest rate of flavonoid release, with the maximum concentration reached within 1 min of dissolution and then decreasing slightly (Figure 7). This increased dissolution rate can be explained by the higher specific surface area of the lyophilized sample compared to other samples, which allows for the fastest release of flavonoid [53]. However, fluctuations in the concentration between 1 min and subsequent points in the dissolution profile for DHQ/LYS_lyo_ may be due to the inefficient distribution of the sample across the volume of the dissolution medium after 1 min.

A slightly lower dissolution rate was observed for the DHQ/LYS_mix_ sample, which reached the maximum release of DHQ after 15 min of the experiment. The concentration of flavonoids in the dissolution medium was somewhat higher for this sample compared to the DHQ/LYS_lyo_. Notably, some of the time points between the DHQ/LYS_lyo_ and DHQ/LYS_mix_ dissolution curves exhibited a lack of significance in concentration. DHQ/LYS_film_ demonstrated a slightly reduced release compared to other obtained objects. For this sample, after 60 min of the experiment, the concentration of flavonoids in the dissolution medium was 3.7 times higher than that of DHQ_raw_. At the same time, DHQ/LYS_mix_ and DHQ/LYS_lyo_ achieved 7.4- and 6.9-fold increases compared to DHQ_raw_.

### 3.10. Limitations and Prospective Further Research

The limitations of the current study include a lack of data on the exact solubility of the obtained objects. This is because when more than 1.0 g of the sample is dissolved in 1 mL of water, the resulting solution has a high viscosity, making further dissolution difficult. The selection of a method for determining the exact solubility will be an area of future work.

A natural extension of this research could involve investigating the impact of phase modification on bioavailability using relevant in vivo models. Based on the objects obtained, it is planned to further develop dosage forms for the treatment of inflammatory processes and wound healing.

## 4. Conclusions

Thus, co-amorphous systems of DHQ and l-lysine in the form of films and lyophilizates were obtained and described using a complex of physicochemical and biological methods of analysis. In this case, the possibility of forming solid film co-amorphous structures for flavonoid compositions with amino acids was shown for the first time. A pronounced increase in solubility was established for the obtained objects compared to the initial flavonoid. The absence of formation of new covalent bonds between the compounds during the formation of such systems was established using the ATR-FTIR and ^1^H NMR methods. The glass transition effect was detected at 64 °C for the obtained films. It is important to note that as a result of studying the cytotoxic properties of the objects, a decrease in cytotoxicity was established during the lyophilization of the mechanical mixture of the initial components.

The obtained objects may be interesting from the point of view of increasing the solubility of natural flavonoids, which solves one of the main problems in developing drugs based on them.

## Figures and Tables

**Table 1:** Prepared modifications.

Modification	Abbreviation	Production Method
Mechanical mixture of DHQ and LYS	DHQ/LYS_mix_	Grinding
Films	DHQ/LYS_film_	Drying
DHQ lyophilizate	DHQ_lyo_	Freeze drying
l-Lysine lyophilizate	LYS_lyo_	Freeze drying
Mechanical mixture lyophilizate	DHQ/LYS_lyo_	Freeze drying

**Table 2:** Results of ^1^H nuclear magnetic resonance analysis.

The Source of the Signal		Chemical Shift Value		Multiplicity of the Signal
Molecule	Proton	Initial Substance	Films	
DHQ	OH5	11.8	-	Singlet
	OH7	10.8	-	Singlet
	OH4′	9.1	-	Singlet
	OH3′	9.0	-	Singlet
	H2′	7.0	7.1	Singlet
	H5′ H6′	6.9	6.9	Singlet
	H6	5.9	5.8	Doublet
	H8	5.8	5.8	Doublet
	H2	5.0	5.0	Doublet
	OH3	4.6	4.3	Singlet
	H3	4.5	4.8	Doublet
l-Lysine	H2	3.4	3.4	Multiplet
	H6	2.9	3.0	Triplet
	H3 H5	1.7	1.8 1.7	Multiplet
	H4	1.4	1.5	Multiplet

**Table 3:** Analysis of variance of the cytotoxicity of dihydroquercetin and l-lysine.

Source of Variation	*df*	*SS*	*MS*	*F*	*F_crit_*	*p*-Value
l-Lysine	1	0.2367488	0.2367488	139.4259802	4.493998478	2.60 × 10^−9^
DHQ	1	0.4392648	0.4392648	258.6915976	4.493998478	2.67 × 10^−11^
Interaction	1	0.4422338	0.4422338	260.4400995	4.493998478	2.54 × 10^−11^
Inside	16	0.0271684	0.0016980			
Total	19	1.1454158				

*df*—the number of degrees of freedom, *SS*—the sum of the squared deviations, *MS*—the variance, *F*—the actual value of the Fisher ratio, and *F_crit_*—the critical value of the Fisher ratio.

## Data Availability

The original contributions presented in this study are included in the article/Supplementary Materials. Further inquiries can be directed to the corresponding author.

## Supplementary Materials

The following supporting information can be downloaded at: https://www.mdpi.com/article/10.3390/pharmaceutics17121528/s1, Figure S1: Attenuated total reflection Fourier transform infrared spectroscopy spectra of different samples; Figure S2: Dependence of the optical density of the CCK-8 solution upon addition of various concentrations of the samples; Figure S3: Dependence of cell survival for various cell lines; Figure S4: Calibration curve for DHQ; Table S1: Standard deviations and RSD for calibration curve for DHQ.

## Abbreviations

The following abbreviations are used in this manuscript:

^1^H NMR	^1^H nuclear magnetic resonance
3T3	Mouse embryonic fibroblast cell line
ANOVA	Analysis of variance
ATR-FTIR	Attenuated total reflection Fourier transform infrared spectroscopy
Caco-2	Immortalized human colorectal adenocarcinoma cell line
CCK-8	Cell counting kit
DHQ	Dihydroquercetin
DHQ_raw_	Dihydroquercetin raw substance
DHQ_lyo_	Dihydroquercetin lyophilizate
DHQ/LYS_mix_	Mechanical mixture of dihydroquercetin and l-lysine
DHQ/LYS_lyo_	Lyophilizate of mechanical mixture of dihydroquercetin and l-lysine
DHQ/LYS_film_	Films of dihydroquercetin and l-lysine
DMEM	Needle-modified Dulbecco’s Modified Eagle Medium
DSC	Differential scanning calorimetry
EDTA	Ethylenediaminetetraacetate
FBS	Fetal bovine serum
HEK293	Immortalized human embryonic kidney cell line
HPLC	High-performance liquid chromatography
HUVEC	Human umbilical vein endothelial cell line
IC50	Half-maximal inhibitory concentration
LYS_raw_	l-lysine monohydrate raw substance
LYS_lyo_	l-lysine lyophilizate
MEM	Minimum essential medium
P/S	Penicillin/streptomycin
SEM	Scanning electron microscopy
TG	Thermogravimetry
XRPD	X-ray powder diffraction
