# Supplementary material for: Co-Amorphous Systems Based on Dihydroquercetin and l-Lysine: Synthesis and Evaluation
Source: Pharmaceutics. 2025 Nov 27;17(12):1528. doi: 10.3390/pharmaceutics17121528 (PMC12736791; doi:10.3390/pharmaceutics17121528)
Supplement: Supplementary file 1 [file pharmaceutics-17-01528-s001.zip › pharmaceutics-3985640-supplementary.pdf]

Co-amorphous systems based on dihydroquercetin and L-lysine: synthesis and evaluation

Artem A. Svotin [1,*], Maria D. Korochkina [1], Anastasia A. Khodyachikh [1], Diana R. Kolesnikova [1], Amir Taldaev [2,3,4], Eduard V. Bocharov [3,4], Alexander V. Dzuban [5], Andrey N. Utenyshev [6], Gennadii V. Shilov [6], Youyan Zeng [7], Bo Li [7], Roman P. Terekhov [1] and Irina A. Selivanova [1]

[1] Nelyubin Institute of Pharmacy, Sechenov First Moscow State Medical University, Trubetskaya Str. 8/2, 119991 Moscow, Russia
[2] Laboratory for the Study of Single Biomacromolecules, Institute of Biomedical Chemistry, Pogodinskaya Str. 10/8, 119121 Moscow, Russia
[3] Laboratory of Biomolecular NMR-Spectroscopy, Shemyakin-Ovchinnikov Institute of Bioorganic Chemistry, Miklukho-Maklaya Str. 16/10, 117997 Moscow, Russia
[4] Research Center for Molecular Mechanisms of Aging and Age-Related Diseases, Moscow Center for Advanced Studies, Kulakova Str. 20/1, 123592 Moscow, Russia
[5] Department of Chemistry, Lomonosov Moscow State University, Leninskiye Gory 1-3, 119991 Moscow, Russia
[6] Federal Research Center of Problems of Chemical Physics and Medicinal Chemistry RAS, Academician Semenov Ave. 1, Chernogolovka, 142432 Moscow region, Russia
[7] Guangdong Metabolic Diseases Research Center of Integrated Chinese and Western Medicine (Institute of Chinese Medicine), MOE Key Laboratory of Glucolipid Metabolic Disorder, Guangdong TCM Key Laboratory for Metabolic Diseases, Guangdong Pharmaceutical University, Wai Huan Dong Road 280, 510006 Guangzhou, China

Supplementary Materials

1.1. ATR-FTIR

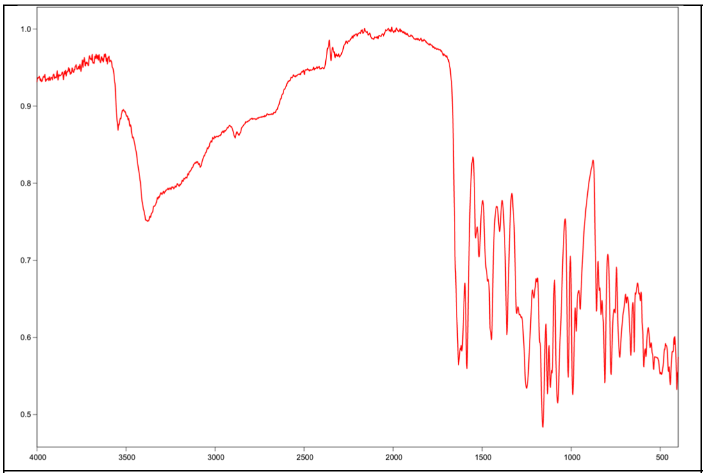

(a)

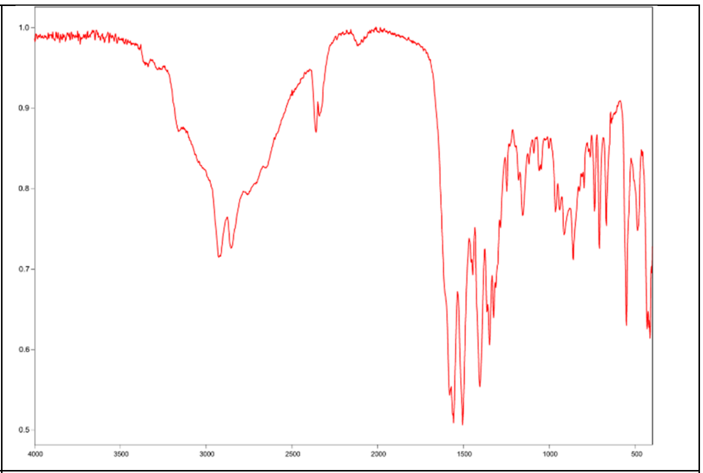

(b)

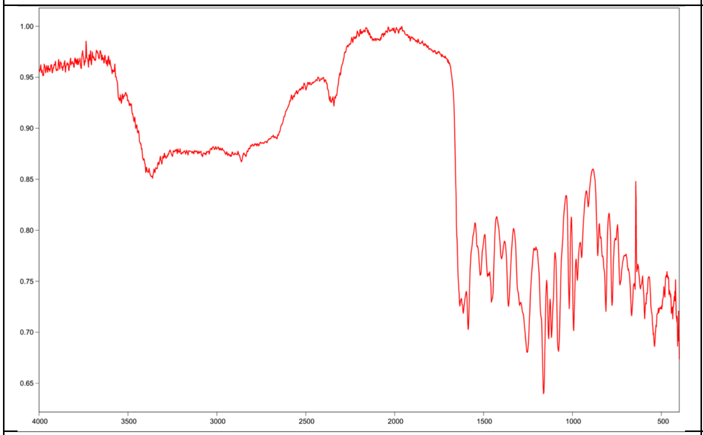

(c)

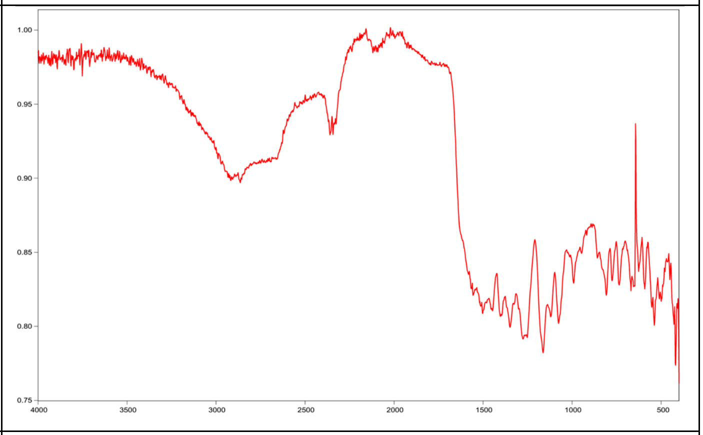

(d)

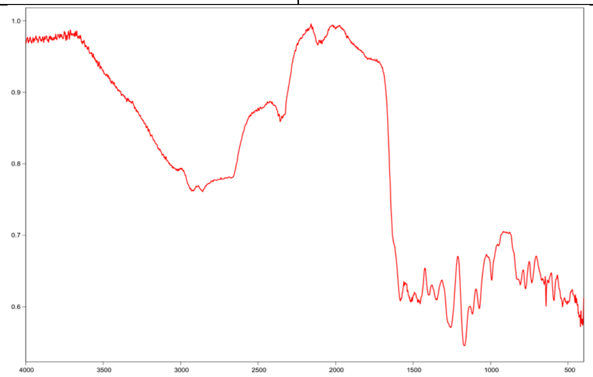

(e)

Figure S1. Attenuated total reflection Fourier transform infrared spectroscopy spectra of different samples: (**a**) dihydroquercetin raw substance; (**b**) L-lysine monohydrate raw substance; (**c**) mechanical mixture of dihydroquercetin and L-lysine; (**d**) lyophilizate of mechanical mixture of dihydroquercetin and L-lysine; (**e**) films of dihydroquercetin and L-lysine

1.2. Blank experiments

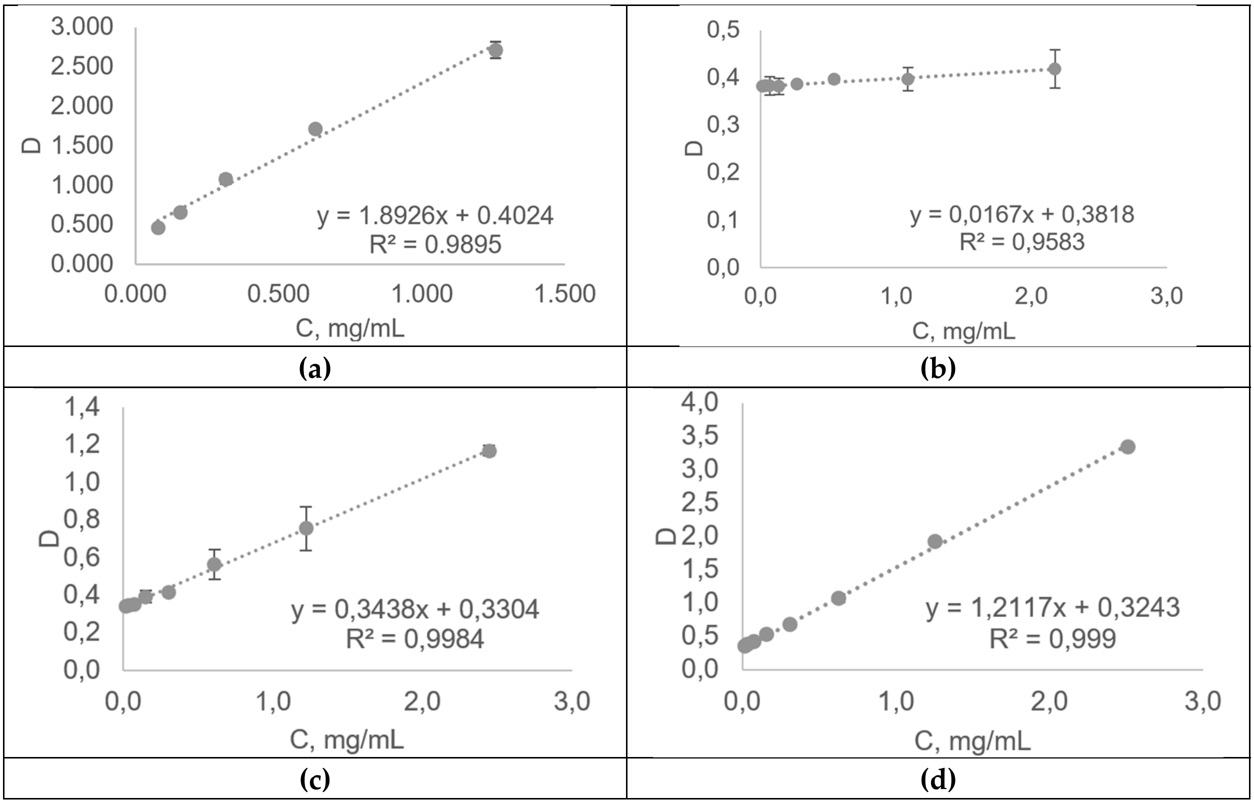

Figure S2. Dependence of the optical density of the CCK-8 solution upon addition of various concentrations of the samples: (**a**) dihydroquercetin raw substance; (**b**) L-lysine lyophilizate; (**c**) mechanical mixture of dihydroquercetin and L-lysine; (**d**) lyophilizate of mechanical mixture of dihydroquercetin and L-lysine

1.3. Cytotoxicity assessment

<table>
<tr><th rowspan="2">Samples</th><th colspan="4">Cell lines</th></tr>
<tr><th>HEK293</th><th>3T3</th><th>HUVEC</th><th>Caco-2</th></tr>
<tr><td>DHQ_{raw}</td>
<td>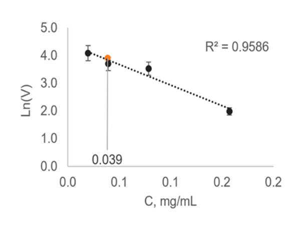</td>
<td>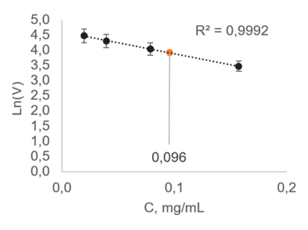</td>
<td>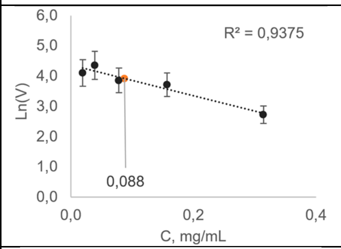</td>
<td>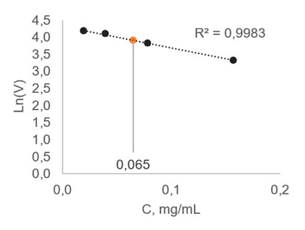</td></tr>
<tr><td>LYS_{lyo}</td>
<td>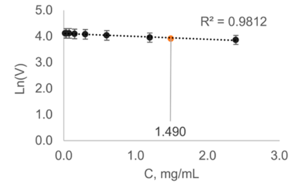</td>
<td>-</td>
<td>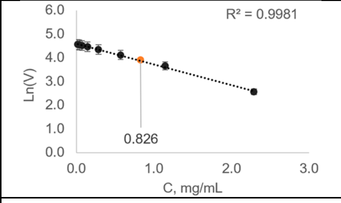</td>
<td>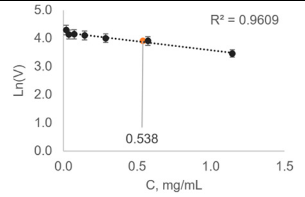</td></tr>
<tr><td>DHQ/LYS_{mix}</td>
<td>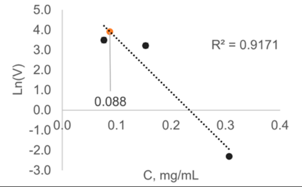</td>
<td>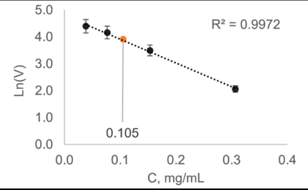</td>
<td>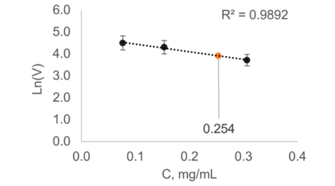</td>
<td>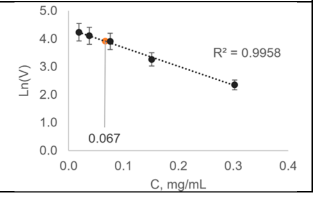</td></tr>
</table>

DHQ/LYS_{lyo}

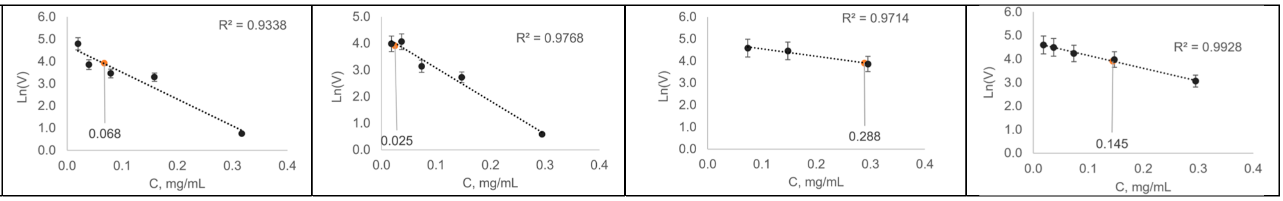

Figure S3. Dependence of cell survival for various cell lines. The orange dot indicates the IC50 value

1.4. HPLC analysis

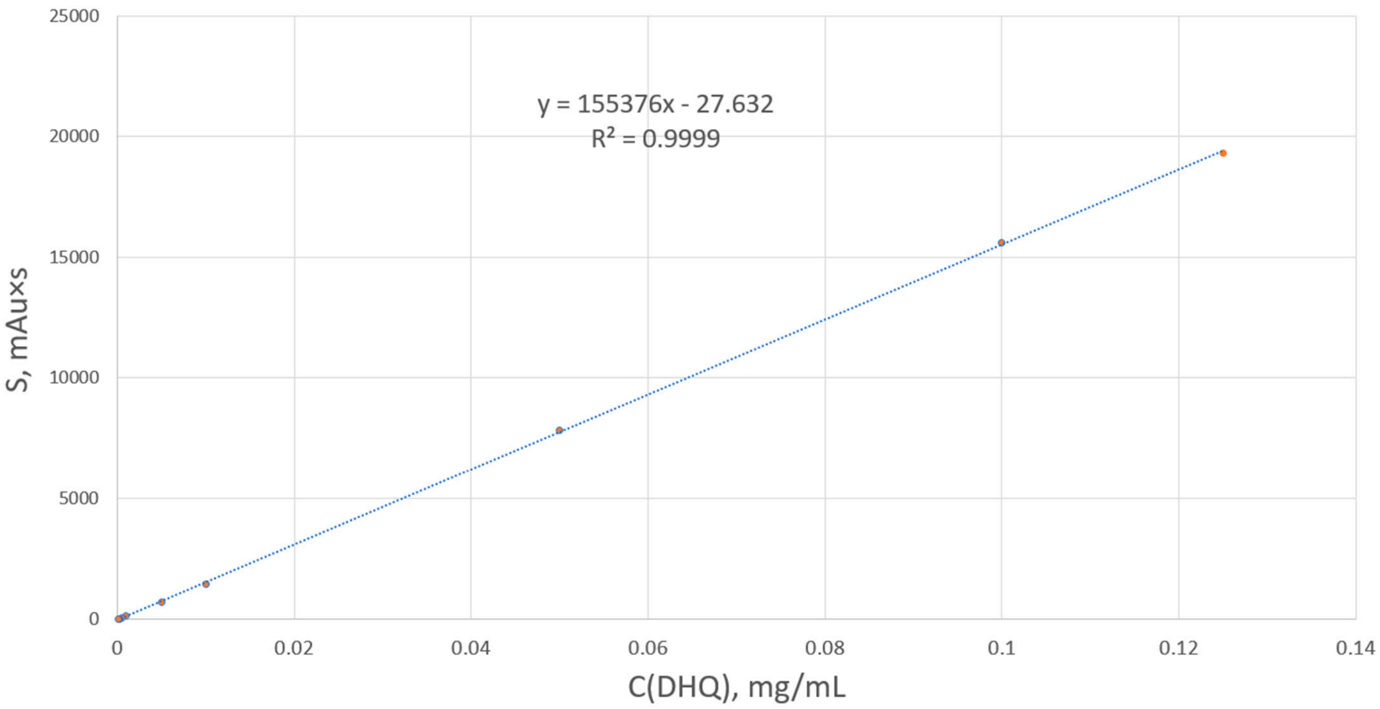

Figure S4. Calibration curve for dihydroquercetin

Table S1. Standard deviations and RSD for calibration curve for dihydroquercetin

C(DHQ), mg/mL	Average square (*n*=3), mAu×s	Standard deviation, mAu×s	RSD, %
0.12500	19304.0500	15.059490	0.078012
0.10000	15596.4200	55.755710	0.357491
0.05000	7811.6450	9.741954	0.124711
0.01000	1463.9390	2.859553	0.195333
0.00500	698.3798	0.749136	0.107268
0.00100	121.4150	1.920734	1.581957
0.00050	58.5554	1.055350	1.802310
0.00025	29.3423	0.479286	1.633433
0.00010	14.1327	0.163549	1.157241
